# No Enhancement of 24-Hour Visuomotor Skill Retention by Post-Practice Caffeine Administration

**DOI:** 10.1371/journal.pone.0129543

**Published:** 2015-06-08

**Authors:** Sara J. Hussain, Kelly J. Cole

**Affiliations:** Department of Health and Human Physiology, University of Iowa, Iowa City, Iowa, United States of America; Tokai University, JAPAN

## Abstract

Caffeine is widely consumed throughout the world and appears to indirectly facilitate learning and memory through effects on attention and motivation. Animal work indicates that post-training caffeine administration augments inhibitory avoidance memory, spatial memory, and object memory. In humans, post-training caffeine administration enhances the ability to discern between familiar images and new, similar images. However, the effect of post-training caffeine administration on motor memory has not been examined. Therefore, we tested two groups of low caffeine consumers (average weekly consumption ≤500 mg) in a double-blind, placebo-controlled study involving acquisition of a continuous isometric visuomotor tracking skill. On Day 1, subjects completed 5 blocks (150 repetitions) of training on the continuous isometric visuomotor skill and subsequently ingested either 200 mg of caffeine or placebo. On day 2, subjects completed an additional 5 blocks of training. Day 1 mean performance and performance variability were both similar between groups, suggesting that both groups acquired the motor skill similarly. For mean performance on Day 2, patterns of re-learning, mean performance learning magnitudes, mean performance learning rates, and mean performance retention magnitudes were all similar between groups. For performance variability on Day 2, there was a small trend towards increased variability in the caffeine group during re-learning, but performance variability learning magnitudes and performance variability retention magnitudes did not differ between groups. Because motor skill acquisition can also be conceptualized as a reduction in performance variability, these results suggest that there may be a small negative effect of post-practice caffeine administration on memory of a newly-learned visuomotor skill. Overall, we found no evidence to suggest that post-training caffeine administration enhances 24-hour retention of a newly-learned continuous visuomotor skill, and these results support the notion that memory-enhancing effects of post-training caffeine ingestion may be task-specific.

## Introduction

Caffeine is the most widely consumed stimulant in the world [1], and its behavioral and cognitive effects have been well-studied. In the motor domain, caffeine ingestion decreases reaction times [2], but reduces motor steadiness. For example, a single dose of 200–300 mg of caffeine increases whole-arm resting tremor [3,4], which could potentially interfere with the performance of motor skills.

Caffeine also may improve learning and memory, although it is difficult to exclude increased arousal and motivation at the time of learning as the underlying cause of these improvements [5,6]. However, recent work indicates that caffeine directly impacts the neural mechanisms underlying learning and memory. Acute administration of caffeine immediately *after* task exposure improves both 24-hour inhibitory avoidance memory and 24-hour spatial memory in rodents [7,8,9]. In contrast, caffeine administration after exposure to a novel environment does not enhance memory for the new environment [8], suggesting that caffeine may not improve all forms of memory. Recent work in humans showed that ingestion of a single 200 mg dose of caffeine immediately after exposure to visual images enhanced subjects’ ability to discriminate between those images and lure images (i.e., images that resembled those presented initially) one day after initial exposure [10]. Because caffeine was given immediately after learning in this study and in the aforementioned animal work [7,8,9] it appears that a single dose of caffeine may stabilize the neural representation of newly-acquired information.

Few studies have directly examined the effects of caffeine on motor memory, and these have produced conflicting results. When caffeine was ingested prior to conditional motor skill learning, the rate of learning across three consecutive days increased but the final amount of learning was unaffected [11]. In contrast, when caffeine was ingested 60 minutes prior to recall of a newly-learned motor sequence, memory was impaired [12]. In these studies, caffeine was administered prior to testing; thus, the attentional, motivational, and psychomotor effects of caffeine cannot be ruled out. These potentially confounding effects can be avoided by administering caffeine immediately *after* motor practice, and examining retention of the newly-learned skill 24 hours later after the acute effects of caffeine have dissipated. In this way, changes in performance one day later can be attributed to alteration of offline processes underlying motor memory stabilization. However, we are unaware of any reports using such an experimental design in the context of motor learning.

We therefore aimed to determine if a single dose of caffeine immediately after visuomotor skill acquisition alters 24-hour retention of the skill. To answer this question, we performed a double-blind, placebo-controlled trial in healthy, young individuals who normally consume low levels of caffeine. Based on recent findings that post-training caffeine administration improves memory processes in humans [10] we predicted that subjects who ingest caffeine immediately after practicing a novel continuous visuomotor skill would demonstrate better 24-hour retention of the skill than those who ingested placebo immediately after practice. However, we found no evidence that post-practice caffeine administration improved retention of the continuous visuomotor skill.

## Materials and Methods

### Participants

We recruited 26 subjects for participation in this study. Subjects were assigned to either the caffeine or placebo group to ensure similar distributions of age, sex and average weekly caffeine consumption between groups (see Table 1). Subjects were free of neurological, orthopedic, or cardiovascular disorders and were not taking any medications that act on the central nervous system. All subjects reported right hand dominance and consumed less than 500 mg of caffeine during an average week. We recruited low caffeine consumers because these individuals may be more sensitive to caffeine ingestion, which should strengthen our ability to detect an effect of caffeine on motor memory. However, it is important to note that results from low caffeine consumers may not generalize to individuals who consume higher levels of caffeine (James 2014).

**Table 1:** Subject characteristics.

Measure	Caffeine Group (mean ± SEM)	Placebo Group (mean ± SEM)	t-value (df = 24)	p-value
Avg. Weekly Caffeine Consumption	160.8 ± 39.3 mg	97.5 ± 32.7 mg	1.332	0.195
Age	22.9 ± 0.9 yrs	24 ± 0.8 yrs	-1.05	0.304
Sex	6 M, 8 F	6 M, 6 F	—	—
Sleep Quality	4.1 ± 0.2	4.1 ± 0.2	-0.043	0.966
Sleep Duration	7.2 ± 0.4 hrs	7.3 ± 0.4 hrs	-0.076	0.940

Caffeine consumption levels were estimated via paper and pencil survey. Subjects were asked to indicate how many servings of commonly-ingested caffeine substances they consume during an average week, as well as any other substances they regularly consume which might contain caffeine. Subjects were also asked to indicate the number of hours they slept overnight between Day 1 and 2 testing, and rated their sleep quality using a 5-point scale (1 = poor sleep quality; 5 = excellent sleep quality).

Subjects were instructed to abstain from caffeine for 12 hours prior to Day 1 testing until the end of Day 2 testing. Subjects also were instructed to abstain from alcohol intake throughout the study due to ethanol’s detrimental effects on long-term potentiation-like plasticity [13], which is thought to be critical for motor memory [14]. In addition, Day 1 testing was performed before 4 pm to minimize sleep disruption due to caffeine intake. All subjects provided their written informed consent. Study procedures were approved by the Institutional Review Board at the University of Iowa and conformed to the standards set forth by the Declaration of Helsinki.

### Experimental Design

**Fig 1:**
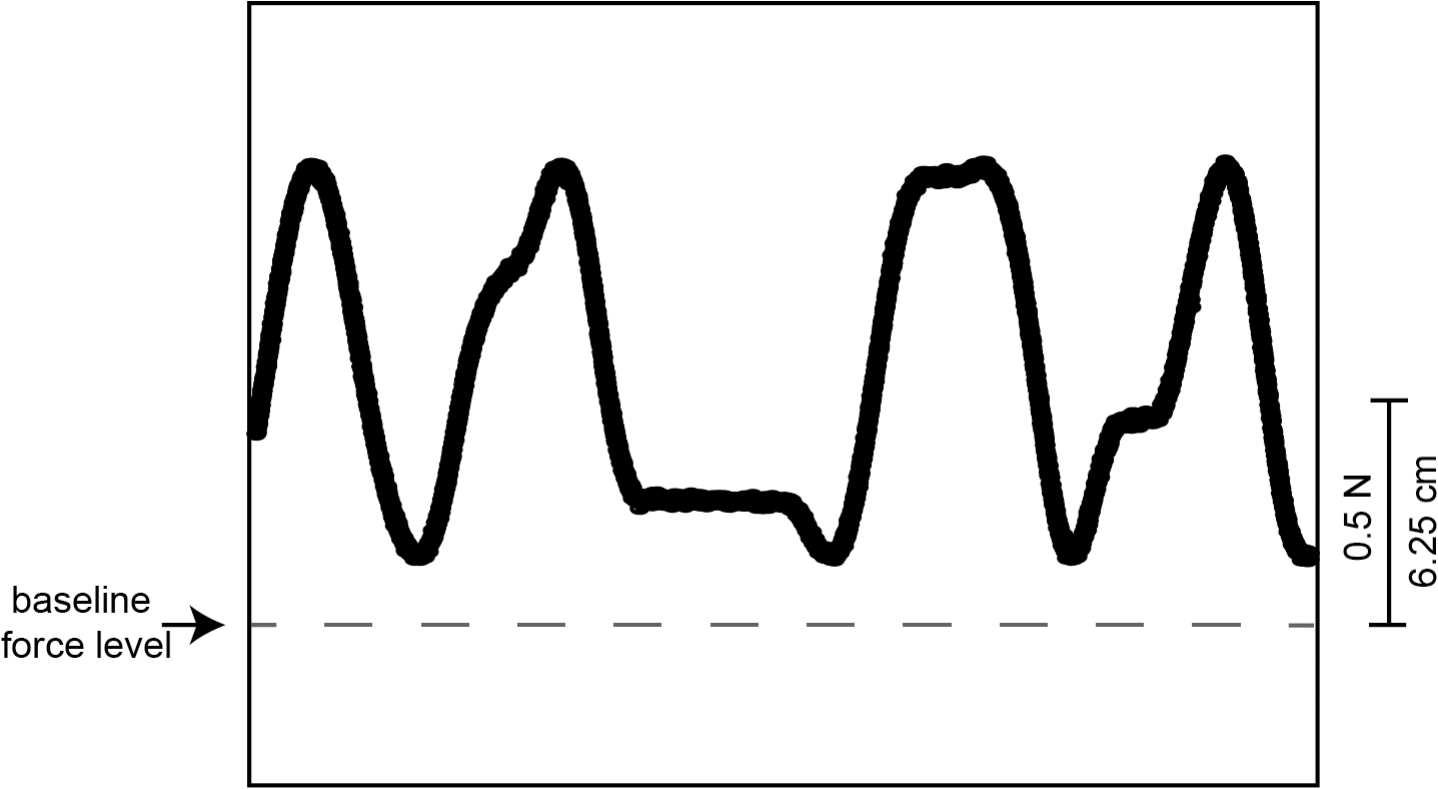
Image of the complex waveform used in the CIVTT. Subjects were instructed to produce a time-varying abduction force by pressing on a small force transducer using their index finger in order to track the complex waveform. The application of force onto the transducer drove an oscillographic display in which the cursor moved automatically from left to right at a rate of 5.9 cm/sec.

On Day 1, subjects reported to the laboratory, and only those who reported abstinence from caffeine and alcohol were invited to participate. For this study, we developed a continuous isometric visuomotor tracking task (CIVTT) that requires precise control of index finger abduction force. During motor practice, subjects were seated at a small console. The right hand was placed palmar side down on a flat surface, with the webbing between the index finger and thumb braced against a vertical steel rod. The medial border of the hand and forearm was blocked in place using sandbags, and a small sandbag was laid on top of the forearm to restrain motion further. The radial side of the distal phalanx of the index finger contacted a small force transducer with a round surface (diameter = 3.3 cm; ATI Nano, Assurance Technologies, Garner NC) covered with fine-grain sandpaper and mounted on a rigid frame. Subjects were instructed to abduct their index finger against the force transducer to generate a force that drove an oscillographic display on a flat computer monitor in order to control the vertical movement of a cursor. The cursor scrolled automatically from left to right at 5.9 cm/sec. During testing, a single stationary complex waveform was statically displayed on the computer screen (Fig 1), and this waveform was visible during all trials. To successfully track the waveform, subjects had to produce a time-varying abduction force. The cursor moved 1 cm vertically per 0.08 N of abduction force, with a maximum waveform height of 12.5 cm (i.e., peak of waveform equivalent to 1.02 N of abduction force). The waveform template was 26 cm long, each trial lasted for 4.4 seconds, and subjects rested for a minimum of 1.6 seconds between trials. In general, most subjects rested for 2–4 seconds between trials and therefore moved through each practice block in a self-paced manner. Subjects completed 5 blocks of 30 trials each, and rested for 2 minutes between blocks but were allowed an additional 1–2 minutes of rest between blocks if requested. During each trial of the CIVTT, subjects received continuous visual feedback of the cursor position, and both the tracking trajectory from the previous trial and the complex waveform were displayed on the computer screen after each trial. Subjects could visually compare their tracking trajectory to the shape of the complex waveform after each trial. After all training blocks were complete, subjects ingested (with water) either a 200 mg capsule of caffeine anhydrous or a visually-identical placebo capsule. Subjects were then free to leave the laboratory and were reminded to abstain from caffeine and alcohol until the next testing session.

Approximately 24 hours later, subjects returned to the laboratory. All subjects reported abstinence from caffeine and alcohol between sessions. Subjects then indicated their sleep duration and quality. Sleep quality was measured using a 5-point scale, where 1 indicated poor sleep quality and 5 indicated excellent sleep quality. Subjects then completed 5 blocks of 30 trials each of the CIVTT in the same manner as day 1.

### Data Collection and Analysis

DATAPAC 2.0 software (RUN Technologies, Mission Viejo CA) was used to collect force signals generated by index finger abduction. Force signals applied perpendicular to the plane of the transducer were sampled at 1000 Hz and data were stored for offline analysis.

The main outcome measure for this study was root mean square error (RMSE; Schmidt and Lee 1988) of the target waveform and the tracking force-time trajectory generated by index finger abduction force. Here, RMSE measures the discrepancy between perfect performance and actual performance. Smaller RMSE values indicate better performance (less tracking error), whereas larger RMSE values indicate poorer performance (more tracking error). RMSE was calculated for each trial of each block using custom-written MATLAB routines (TheMathWorks, Natick MA). We calculated mean performance from trial-by-trial RMSE values within each block, and as a secondary measure of skill acquisition [15], we also determined each subject’s performance variability by calculating the trial-by-trial standard deviations for each block.

We also calculated learning rates using mean performance values. We determined the mean performance learning rates on days 1 and 2 by fitting each subject’s data with an exponential function. For each day, we first averaged each subject’s trial-by-trial data into 15 consecutive bins of 10 trials each. Then, we fit a single exponential function to each subject’s Day 1 and Day 2 data using the least squares method. The general form of the exponential function was:

$$a+be^{-cn}$$

where *a* is the performance asymptote, *b* is the change in performance from bin 1 to performance asymptote, *n* is the amount of practice, and *c* is the rate constant. The rate constant was defined as the proportion of mean performance reduced per unit time and was taken as the measure of learning rate. Smaller rate constant values indicate slower skill acquisition and larger rate constant values indicate faster skill acquisition. In one case where the *b* parameter was negative (i.e., exponential fit showed performance losses during practice), the rate constant value was set at 0. Additionally, we did not include performance variability learning rates in our overall analyses due to poor fits of the exponential models with binned standard deviation data (averaged R^2^ value for exponential fits for binned standard deviation data = 0.56 ± 0.04 [SEM]).

For both mean performance and performance variability, we calculated learning magnitudes for each day using the following formula:

$$\frac{\left(RMSE_{Block1}-RMSE_{Block5}\right)}{RMSE_{Block1}}*100$$

Here, learning magnitudes represent the percentage of either mean tracking error (for mean performance learning magnitudes) or variability in tracking error (for performance variability learning magnitudes) that subjects reduced by the end of testing on each day. Finally, we calculated retention magnitudes for mean performance and performance variability by dividing Block 5 Day 1 values by Block 1 Day 2 RMSE values and multiplying by 100. Hence, retention magnitudes of 100% represent complete retention of practice-induced gains in mean performance or reductions in variability, whereas retention magnitudes greater than 100% or less than 100% represent offline gains or losses, respectively.

### Statistical Analysis

We tested for group differences in age, average weekly caffeine consumption, sleep quality and sleep duration using unpaired t-tests. Separate group x block analysis of variance (ANOVA), with repeated measures on factor block and Huynh-Feldt correction, were performed on mean performance and performance variability values for each day. Group x day ANOVA, with repeated measures on factor day, were used to examine differences in mean performance learning rates, mean performance learning magnitudes and performance variability learning magnitudes. We also performed unpaired t-tests to assess group differences in mean performance and performance variability retention magnitudes. Finally, Pearson’s correlations were used to examine relationships between average weekly caffeine consumption and mean performance and performance variability learning magnitudes, mean performance and performance variability retention magnitudes, and mean performance learning rates. In the event of significant main effects or interactions, Tukey’s Honestly Significant Difference test was used for post-hoc testing. Alpha was equal to 0.05 for all comparisons.

## Results

Average weekly caffeine consumption levels, age, sleep quality and sleep durations were similar between groups (see Table 1). All subjects reported caffeine abstinence starting 12 hours prior to their first session until the end of session 2, as well as alcohol abstinence for the duration of the study. No subject reported an adverse reaction to ingestion of either the caffeine or placebo capsule.

### Mean Performance

**Fig 2:**
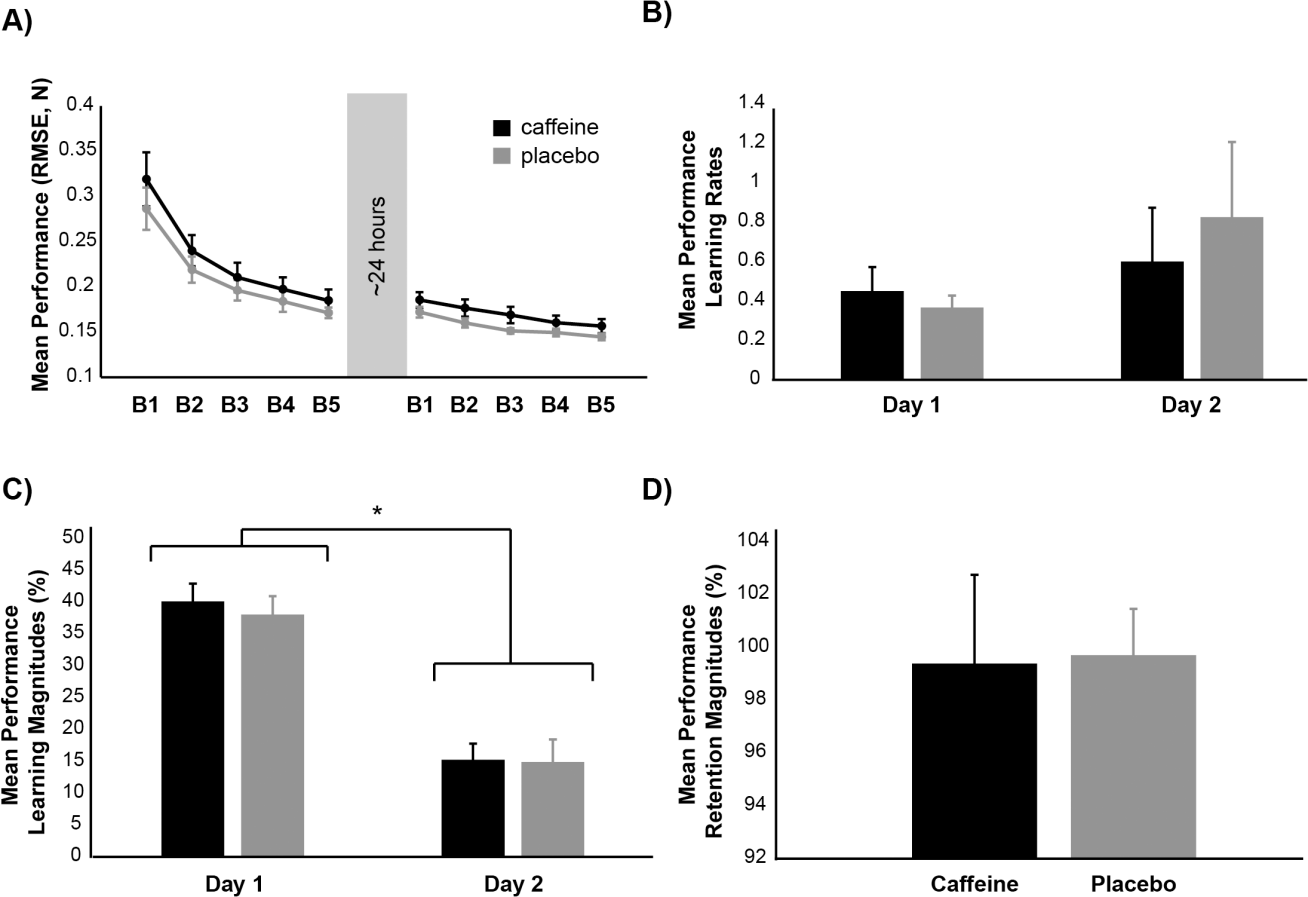
Group averaged mean performance on Days 1 and 2. A) Group averaged mean performance across all testing blocks. B) Mean performance learning rates. C) Mean performance learning magnitudes. D) Mean performance retention magnitudes. On Days 1 and 2, both groups significantly improved mean tracking performance with practice, and mean performance learning magnitudes were larger on Day 1 than Day 2. However, there were no significant differences between groups for any mean performance measure. Black represents the caffeine group, whereas grey represents the placebo group. B1 = block 1, B2 = block 2, B3 = block 3, B4 = block 4, B5 = block 5. Error bars, ± 1 SEM.

On day 1, subjects improved their mean tracking performance across blocks (Fig 2A, main effect of block, F_4,96_ = 74.765, adjusted p<0.001, ɳ^2^_partial_ = 0.757), and the pattern of this improvement did not differ between groups (no main effect of group, F_1,24_ = 0.799, p = 0.380; no group x block interaction, F_4,96_ = 0.529, adjusted p = 0.536). Post-hoc testing indicated that subjects in both groups improved their mean tracking performance over time (post-hoc comparisons of block 1 vs. blocks 2–5, p<0.001 for all) and that mean performance plateaued during block 4 (post-hoc comparison of block 4 vs. block 5, p = 0.529). On day 2, subjects again improved their mean performance as practice progressed (main effect of block, F_4,96_ = 27.244, adjusted p<0.001, ɳ^2^_partial_ = 0.532), and again these performance gains were similar between groups (no main effect of group, F_1,24_ = 2.401, p = 0.134; no group x block interaction, F_4,96_ = 0.444, p = 0.693). Post-hoc testing again revealed that subjects improved their mean tracking performance over time (post-hoc comparisons of block 1 vs. blocks 2–5, p<0.01 for all), and that mean performance plateaued during block 4 (post-hoc comparison of block 4 vs. block 5, p = 0.641).

Subjects in both groups improved their average tracking performance at similar rates on Days 1 and 2 (Fig 2B, no main effect of day, F_1,24_ = 1.678, p = 0.208; no main effect of group, F_1,24_ = 0.099, p = 0.756; no group x day interaction, F_1,24_ = 0.431, p = 0.518). Exponential fits for calculation of Day 1 learning rates yielded an R^2^ value of 0.94 ± 0.01 (SEM), but fits for Day 2 produced an average R^2^ value of 0.64 ± 0.05 (SEM) due to greater within-subject bin-by-bin variability. Removal of one data point that met our criteria for outliers (i.e., 3 SD outside of the mean) did not alter the pattern of findings for learning rates. Mean performance learning magnitudes were greater on Day 1 than Day 2 (Fig 2C, main effect of day, F_1,24_ = 124.173, p<0.001, ɳ^2^_partial_ = 0.838; post-hoc comparison of day 1 vs. day 2, p<0.001), and did not differ between groups (no main effect of group, F_1,24_ = 0.127, p = 0.725; no group x day interaction, F_1,24_ = 0.159, p = 0.694). Mean performance retention magnitudes also did not differ between groups (Fig 2D, t_24_ = -0.081, p = 0.936). Thus, subjects in both groups showed similar mean performance learning rates, mean performance learning magnitudes, and mean performance retention magnitudes.

**Fig 3:**
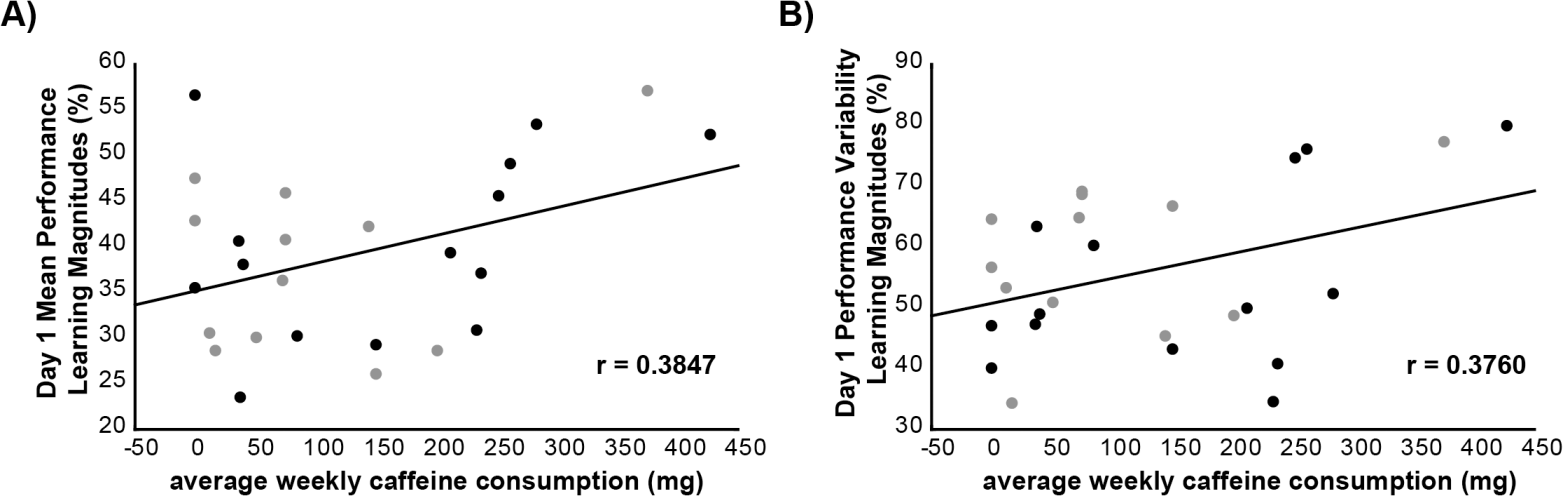
Correlation between average weekly caffeine consumption and day 1 learning magnitudes. A) Correlation between average weekly caffeine consumption and day 1 mean performance learning magnitudes. B) Correlation between average weekly caffeine consumption and day 1 performance variability learning magnitudes. Black dots depict subjects in the caffeine group, and grey dots depict subjects in the placebo group.

Pearson’s correlations revealed no significant relationships between average weekly caffeine consumption, Day 2 mean performance learning magnitudes, Day 1 and 2 mean performance learning rates, or mean performance retention magnitudes (p>0.350 for all). However, we detected a marginally significant positive relationship between average weekly caffeine consumption and Day 1 mean performance learning magnitudes (Fig 3A, r = 0.3847, p = 0.052).

### Performance Variability

**Fig 4:**
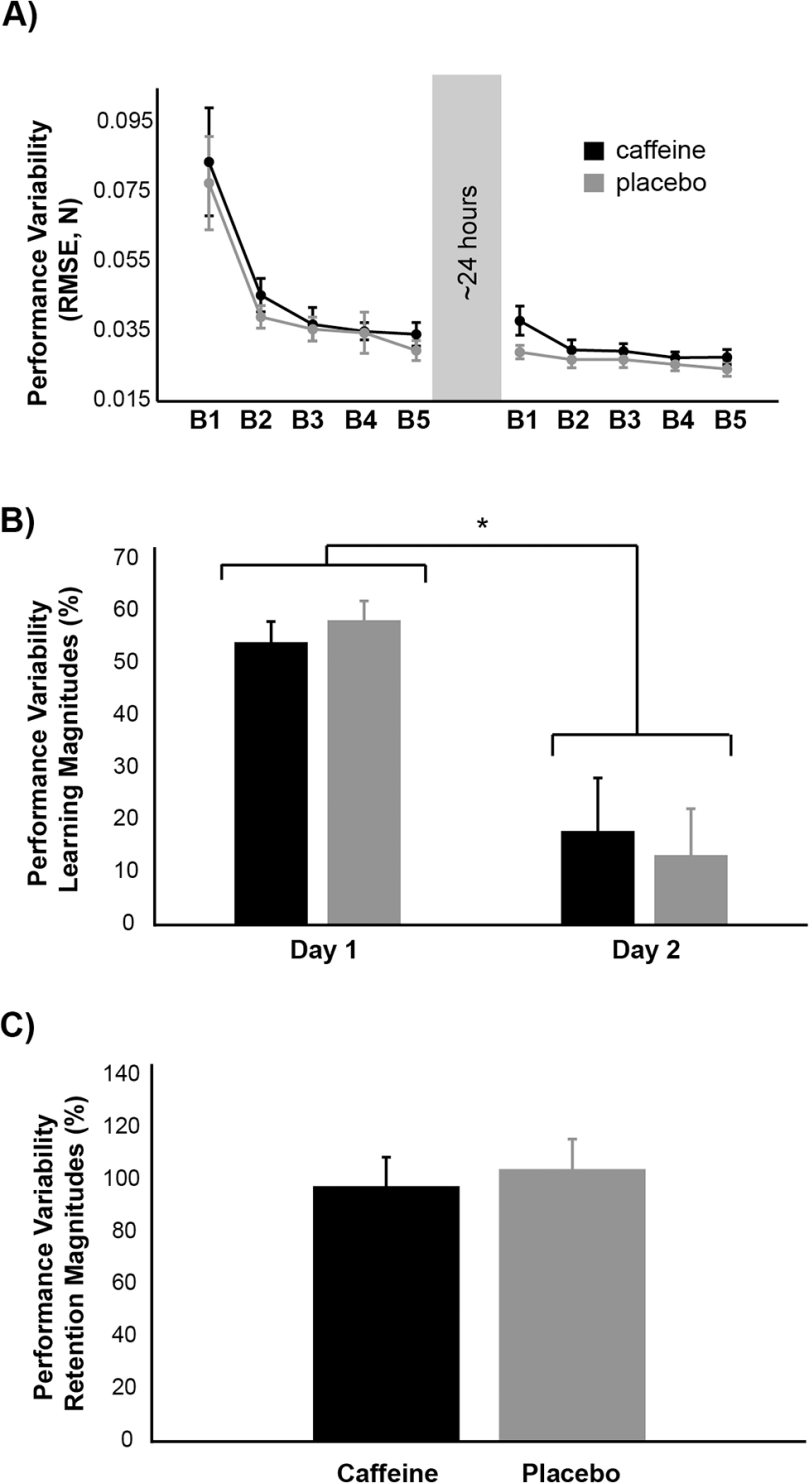
Group averaged performance variability on Days 1 and 2. A) Group averaged performance variability across all testing blocks. B) Performance variability learning magnitudes. C) Performance variability retention magnitudes. On Days 1 and 2, both groups significantly reduced their performance variability. Performance variability learning magnitudes were larger on Day 1 than on Day 2, but there was no difference between groups for performance variability retention magnitudes. Black represents the caffeine group, whereas grey represents the placebo group. B1 = block 1, B2 = block 2, B3 = block 3, B4 = block 4, B5 = block 5. Error bars, ± 1 SEM.

Subjects in the caffeine and placebo groups reduced their performance variability with practice on Day 1 (Fig 4A, significant main effect of block, F_4,96_ = 25.058, p<0.001, ɳ^2^_partial_ = 0.511) and there were no differences in the pattern of this reduction between groups (no main effect of group, F_1,24_ = 0.270, p = 0.608; no group x block interaction, F_4,96_ = 0.113, p = 0.833). Post-hoc testing on the main effect of block showed that the reduction in performance variability plateaued during block 2 (post-hoc comparisons of block 1 vs. blocks 2–5, p<0.001; block 2 vs. blocks 3–5, p>0.350). On day 2, both groups continued to reduce performance variability with practice (significant main effect of block, F_4,96_ = 3.965, p = 0.011, ɳ^2^_partial_ = 0.142), although subjects in the caffeine group tended to show greater within-block performance variability than subjects in the placebo group (trend towards main effect of group, F_1,24_ = 3.493, p = 0.074; no group x block interaction, F_4,96_ = 0.906, p = 0.444). Post-hoc testing on the main effect of block revealed that reduction in performance variability again plateaued during block 2 (post-hoc comparisons of block 1 vs. blocks 2–3, p>0.06; block 1 vs. blocks 4–5, p<0.01; block 2 vs. blocks 3–5, p>0.808).

Performance variability learning magnitudes were larger on Day 1 compared to Day 2 (Fig 4B, significant effect of day, F_1,24_ = 28.933, p<0.001, ɳ^2^_partial_ = 0.547; post-hoc comparison of day 1 vs. day 2 learning magnitudes, p<0.001) and there were no differences in performance variability learning magnitudes between groups on either day (no main effect of group, F_1,24_ = 0.001, p = 0.977; no group x day interaction F_1,24_ = 0.345, p = 0.562). There was also no difference between groups in performance variability retention magnitudes (Fig 4C, t_24_ = -0.429, p = 0.672). Overall, subjects in both groups exhibited similar performance variability learning magnitudes and performance variability retention magnitudes.

Pearson’s correlations revealed no significant relationships between average weekly caffeine consumption, Day 2 performance variability learning magnitudes and performance variability retention magnitudes (p>0.190 for all), but there was a nearly significant positive relationship between average weekly caffeine consumption and Day 1 performance variability learning magnitudes (Fig 3B, r = 0.3760, p = 0.058).

## Discussion

To our knowledge, this is the first study to examine the effects of post-training caffeine administration on memory of a visuomotor skill, and one of only two studies examining post-training caffeine ingestion in the context of learning and memory in humans [10]. We found no evidence that post-practice caffeine ingestion improved 24-hour retention of a continuous visuomotor skill. Specifically, on Day 1, prior to any caffeine or placebo administration, subjects in both groups demonstrated similar improvements in mean tracking performance and similar reductions in performance variability. Day 1 learning rates and magnitudes were also similar, indicating that there was no systematic bias in motor performance or learning ability at baseline. On day 2, subjects who had ingested 200 mg of caffeine immediately after Day 1 skill acquisition demonstrated similar retention magnitudes, re-learning magnitudes and re-learning rates compared to those who had ingested placebo after Day 1 skill acquisition. However, we found that subjects in the caffeine group showed greater within-subject performance variability than those in the placebo group on Day 2, although this effect did not reach significance. Because motor learning can also be assayed as a reduction in performance variability with practice [15], this small trend suggests that post-practice caffeine administration may slightly negatively impact motor memory. Finally, we also detected near-significant positive relationships between average weekly caffeine consumption and Day 1 mean performance learning magnitudes and Day 1 performance variability learning magnitudes. At first, the correlational results may appear to contradict our overall findings, but it is important to note that any benefits of regular caffeine use detected using performance measures (such as the learning magnitudes examined here) do not necessarily extend to retention [16].

Our findings contrast with a recent study by Borota and colleagues [10]. These investigators reported that ingesting 200 mg of caffeine immediately after studying visual images improved subjects’ ability to discriminate between the studied images and lure images (i.e., images that resembled the images that were previously studied). Animal studies utilizing post-training caffeine administration have demonstrated augmented inhibitory avoidance memory [7,8] and improved spatial memory [9]. In contrast, retention of habituation learning was unaffected by post-training caffeine administration [8]. Angelucci and colleagues suggested that the effects of caffeine on memory cannot be generalized to all forms of learning and that such effects are probably task-dependent. Long-term potentiation (LTP) is a well-accepted biological substrate for learning and memory [17], so one possible explanation is that the task-dependent effects of caffeine on memory are related to differences in the forms of LTP underlying the memory of each task. For example, differences exist in hippocampal LTP even between basal and apical dendrites; LTP at apical dendrites becomes resistant to reversal much faster than LTP at basal dendrites does [18]. Likewise, it is reasonable to consider that different learning tasks might rely on LTP-like processes with different properties. Such differences may mediate caffeine’s apparently task-dependent effects.

Genetic variation also may have contributed to the discrepancy between our results and those from Borota and colleagues [10], although this seems unlikely. Polymorphisms in the genes encoding brain-derived neurotrophic factor (BDNF) and catechol-*O*-methyl transferase (COMT) can affect motor learning. For example, presence of the BDNF polymorphism impairs motor learning and cortical plasticity [19,20,21,22,23], and presence of the COMT polymorphism enhances certain types of motor learning [24] and may also influence cognitive function [25,26]. Thus, it is possible that the groups tested in our study versus the Borota study had different distributions of individuals with pro-learning and pro-memory genotypes, but because neither of these studies tested for polymorphisms in the BDNF or COMT gene, we cannot be sure if genetic variability can explain our contrasting findings. Additionally, in our study, individuals with pro-learning genotypes may not have been equally distributed between groups, but if this was the case we would expect to observe significant differences between groups during Day 1 skill acquisition prior to any caffeine or placebo administration. Indeed, subjects in both groups performed very similarly during Day 1 skill acquisition, so we do not think differences in genetic variability between groups significantly impacted our findings.

One motivation for this study was that caffeine administration might improve motor memory through adenosine receptor antagonism. Long-term potentiation-like plasticity is thought to underlie motor learning [14,27,28,29], and may even be critical for motor skill retention [14], so any intervention that alters the induction and maintenance of LTP-like changes in human cortex might also alter memory [30]. Electrophysiological studies have demonstrated that supra-physiological concentrations of adenosine can prevent the development of LTP in hippocampal slices [31,32], potentially through activation of A1 receptors [31,33]. The adenosine receptor agonist 2-chloradenosine inhibits the induction of LTP in the dentate gyrus in vivo [34], and endogenous adenosine also exerts a tonic inhibitory influence on the development of LTP in rat hippocampal slices [35]. Because the effects of moderate caffeine consumption are primarily mediated by adenosine receptor antagonism [5,36], A1 receptor antagonism via caffeine could attenuate adenosine’s inhibitory influence on LTP. In support of this idea, theophylline (a non-specific adenosine receptor antagonist that is chemically similar to caffeine) facilitates hippocampal LTP [37]. The task tested here (CIVTT) relies heavily on visuomotor integration, which involves fronto-parietal circuits [38]. Frontal and parietal cortex have a moderate density of A1 receptors [39], so we hypothesized that caffeine would antagonize A1 receptors in these brain regions, leading to facilitation and stabilization of LTP-like changes in frontal and parietal cortex. However, our findings do not support this hypothesis.

We are aware of limitations to the current study. Caffeine withdrawal may develop during caffeine abstinence [40,41]. Caffeine withdrawal produces symptoms such as headache, tiredness/fatigue, depressed mood, difficulty concentrating, and irritability (for review, see [42]) can impair cognitive and motor performance [42,43,44,45,46], and subsequent caffeine ingestion reverses these effects [40,41,44]. If withdrawal reversal was a significant issue in this study, individuals with higher levels of average weekly caffeine consumption should have performed the poorest on day 1. Instead, we found the contrary: these individuals tended to perform the best (see Fig 3). Thus, we do not think that withdrawal reversal impacted our findings. In addition, it is also possible that the motor learning task used here was not sensitive to the memory-enhancing properties of caffeine. Recent work suggests that motor learning relies on the interaction of several different neural mechanisms, including use-dependent plasticity [47,48], operant reinforcement [48,49], error-based learning [50,51] and explicit strategy [52]. Importantly, different motor learning tasks (such as sequence learning, skill learning, and motor adaptation) likely recruit each of these mechanisms to a different extent [53], and the extent to which each mechanism is recruited might affect consolidation patterns. For example, explicit sequence learning shows sleep-dependent consolidation whereas implicit sequence learning does not [54], and there are also differences in brain activation patterns during consolidation depending on whether explicit information is provided during learning [55]. Therefore, it is conceivable that different motor learning tasks could respond distinctly to post-training caffeine administration, and we caution against over-generalization of our results to other forms of motor learning. Future studies should test the effects of post-training caffeine ingestion in other motor learning paradigms.

Previous work examining caffeine’s effects on learning and memory have relied on either self-reporting of caffeine use [4,12] or biochemical assays to confirm abstinence [10]. We did not collect saliva samples to confirm caffeine abstinence. Nevertheless, if subjects in either group did not abstain from caffeine prior to or during testing, this should have increased the between-subject variability in our data set. Yet, an examination of between-subject variability during Day 2 testing (visualized through the error bars in Figs 2A and 4A) reveals that this variability was small, demonstrating that our sample was relatively homogenous in terms of motor performance. Such homogeneity suggests that either 1) caffeine use was well-controlled during abstinence periods and subjects complied with our requests for caffeine abstinence, or 2) caffeine was ingested during abstinence periods, but this ingestion did not produce much between-subject variability. If the latter scenario occurred, then it appears that any undocumented caffeine ingestion between sessions did not significantly alter the homogeneity of our sample, and thus probably did not impact Day 2 performance. In addition, even if subjects did not completely abstain from caffeine prior to or during the study, subjects in the caffeine group still received a large dose of caffeine immediately after motor skill learning on Day 1. In order to match any effect of this post-practice caffeine ingestion, subjects in the placebo group would have needed to consume a large amount of caffeine (approaching 200 mg) immediately after leaving the laboratory on Day 1. Given that subjects in the placebo group consumed, on average, less than 100 mg of caffeine during a normal week, this possibility seems improbable.

Overall, we found no evidence that post-training caffeine administration enhances 24-hour retention of a newly-learned continuous isometric visuomotor tracking skill. These results contrast with other work completed in both humans and animals [7,8,9,10], but are consistent with claims that post-training caffeine administration does not enhance all types of memory [8]. To better characterize any memory-enhancing effects of post-practice caffeine administration, future studies should examine multiple forms of learning and memory.
